# Glucose-regulated protein 78 demonstrates antiviral effects but is more suitable for hepatocellular carcinoma prevention in hepatitis B

**DOI:** 10.1186/s12985-017-0747-z

**Published:** 2017-04-13

**Authors:** Nai Q. Zheng, Zi H. Zheng, Hai X. Xu, Ming X. Huang, Xiao M. Peng

**Affiliations:** 1 Department of Infectious Diseases, the Third Affiliated Hospital, Sun Yat-Sen University, Guangzhou, China; 2 Jinan University Clinic, the First Affiliated Hospital of Jinan University, Guangzhou, China; 3 Center of Infectious Diseases, the Fifth Affiliated Hospital, Sun Yat-Sen University, 52 Meihua East Road, Zhuhai, 519000 Guangdong China

**Keywords:** Hepatitis B virus, Chronic hepatitis B, Hepatocellular carcinoma, Antiviral therapy, Glucose-regulated protein 78

## Abstract

**Background:**

Hepatitis B virus (HBV) is the leading cause of liver cirrhosis and hepatocellular carcinoma in Asia and Africa. Existing antivirals cannot cure HBV or eliminate risk of hepatocellular carcinoma. Glucose-regulated protein 78 (GRP78) can inhibit HBV replication, but promote virion secretion and hepatocellular cancer cell invasion. For these reasons, the overall effect of GRP78 on HBV production and whether to utilize the HBV replication-inhibitory effect of GRP78 up-regulation or the hepatocellular cancer cell invasion-inhibitory effect of its down-regulation were further investigated in order to improve the efficacy of current antiviral therapy.

**Methods:**

GRP78 regulations in HepG2.2.15 cells were conducted by transfections of expressing vector and small interfering RNA, respectively. The changes in HBV replication, hepatitis B e antigen (HBeAg) synthesis and hepatoma cell motility were monitored.

**Results:**

GRP78 overall decreased HBV production due to its HBV replication-inhibitory effect time-dependently overwhelming virion secretion-promoting effect in HepG2.2.15 cells. Unlike the parental cells (HepG2), HepG2.2.15 cells demonstrated decreased expressions of the major genes in the interferon-β1-dependent pathway. Moreover, the expressions of these genes were not affected by GRP78 regulations. However, GRP78 was found to inhibit HBeAg secretion and to increase the retro-transportation of capsid assembly-interfering HBeAg precursor from the endoplasmic reticulum into the cytosol where new viral nucleocapsids formed. Furthermore, GRP78 overexpression promoted wound healing process (the motility) of HepG2.2.15 cells. In contrast, GRP78 knockdown enhanced HBV replication and HBeAg secretion, but they were abolished by entecavir and furin inhibitor, respectively.

**Conclusions:**

GRP78 mainly demonstrates anti-HBV effects, reducing HBV production and HBeAg secretion. With due regard to the hepatocellular cancer invasion risk of the overexpression and the rectifiability of the unpleasant effects of the knockdown, GRP78 down-regulation may be more suitable to serve as an additive strategy to cover the hepatocellular cancer prevention shortage of current antiviral therapy in the future.

## Background

Hepatitis B virus (HBV) is the leading cause of liver-related deaths in Asia and Africa although there are effective vaccines for decades. Persistent existence of HBV replication is an independent risk factor for liver cirrhosis and hepatocellular carcinoma (HCC) [1, 2]. Antiviral therapy based on current options, recombinant interferon (IFN)-α and nucleotide/nucleoside analogs, has markedly reduced the incidence of liver cirrhosis and HCC [3, 4]. However, it hardly eliminates HBV infection and the suppression of viral replication below the limit of detection does not completely prevent HCC development although liver fibrosis can be stopped [4, 5]. For examples, during nucleoside analog lamivudine treatment, the cumulative HCC rate at 5 years is 3% in patients with chronic hepatitis B (CHB) and up to 30% in those with liver cirrhosis, and even for the more effective and lower resistant entecavir (ETV), five year-treatment only reduces HCC risk from 13.7 to 3.7% in patients with CHB or advanced liver fibrosis [6, 7]. As for the cumulative HCC rates at 10 years, the situation is even worse. No matter ETV or lamivudine is used, such rates are up to 29.6 and 53.4% in patients with CHB and liver cirrhosis, respectively [8]. Thus, it remains imperative to search for new therapeutic targets against HBV infection or HCC development.

HBV is a hepatotropic enveloped virus with 3.2-kb partially double-stranded circular viral genome. The viral genome is packaged inside the capsid that is assembled by viral core protein (hepatitis B core antigen, HBcAg) to generate nucleocapsid in cytosol. The viral envelope mainly consists of small-surface protein (hepatitis B surface antigen, HBsAg). Some small surface protein has additional extensions of pre-S2 (middle surface protein) or pre-S1 and pre-S2 (large surface protein) in N-terminus. These proteins are cotranslationally integrated into the endoplasmic reticulum (ER) membrane. While penetrates into the ER lumen, the viral nucleocapsid is packaged with these trans-membrane proteins to generate mature virions [9]. During the life cycle, HBV also synthesized a secretory core protein that finally matures into hepatitis B e antigen (HBeAg). During synthesis, the nascent peptide chain is directed to the ER by a signal sequence, and develops into HBeAg precursor after the signal sequence is removed. HBeAg precursor is further digested by furin in the Golgi apparatus to generate mature HBeAg [10, 11]. HBeAg precursor detained in the ER or undigested by furin is retro-transported to cytosol or expresses on the surface of hepatocytes [12, 13]. HBeAg released into bloodstream serves as an immunotolerance inducer [14–16]. In contrast, HBeAg precursor on the cell surface has chance to initiate antiviral immunity and in cytosol can inhibit HBV replication via formation of capsidation-incompetent capsids with HBcAg [17–19].

Glucose-regulated protein 78 (GRP78/BiP) constitutively expresses in the ER. It is involved in the major functions of the ER by translocating newly synthesized polypeptides across the ER membrane, facilitating the folding and secretion of proteins, targeting misfolded or unnecessary proteins for ER-associated protein degradation, and serving as the ER stress sensor to release cells from the stress conditions [20, 21]. With regard to HBV infection, GRP78 is at least involved in four important processes, viral replication, viral secretion, HBeAg secretion and HBV-related hepatocellular carcinogenesis. GRP78 has been reported to inhibit HBV replication by facilitating HBV transcript degradation via activation of IFN-β1-2′,5′-oligoadenylate synthetase-RNase L pathway [22], to promote virion secretion via guiding posttranslational ER translocation of the HBV large envelope protein [23, 24], to enhance the retro-transportation of HBeAg precursor from the ER into cytosol [12], which may affect HBeAg secretion, and to facilitate the migration and invasion of HCC via reduction of ER stress-induced apoptosis, expression on cell-surface or conferring drug resistance [25–28]. The effect of HBV replication inhibition suggest that GRP78 up-regulation is of therapeutic potential for chronic HBV infection, but the rest effects on promoting virion secretion and HCC invasion argue against the above potential. Those contradictory effects let us not know the overall effect of GRP78 on HBV production and how to make a choice to utilize the anti-HBV effect of up-regulation or the cancer-preventing effect of down-regulation of GRP78 in the future.

In this study, the overall effect of GRP78 was found to decrease HBV production due to its HBV replication-inhibitory effect time-dependently overwhelming virion secretion-promoting effect in HepG2.2.15 cells. It also inhibited HBeAg secretion via enhancing the retro-transportation of HBeAg precursor. However, GRP78 overexpression significantly increased the motility of HepG2.2.15 cells. In contrast, GRP78 knockdown accumulated intracellular HBV DNA and increased HBeAg secretion, but fortunately abrogated by co-treatment with ETV and furin inhibitor hex-D-arginine (D6R), respectively. These findings have clarified the overall effect of GRP78 on HBV replication and have many implications for strategy searches in order to cover the HCC prevention shortage of current antiviral therapy in the future.

## Results

### GRP78 overwhelms its viral secretion-promoting effect to suppress HBV production in HepG2.2.15 cells

**Fig. 1:**
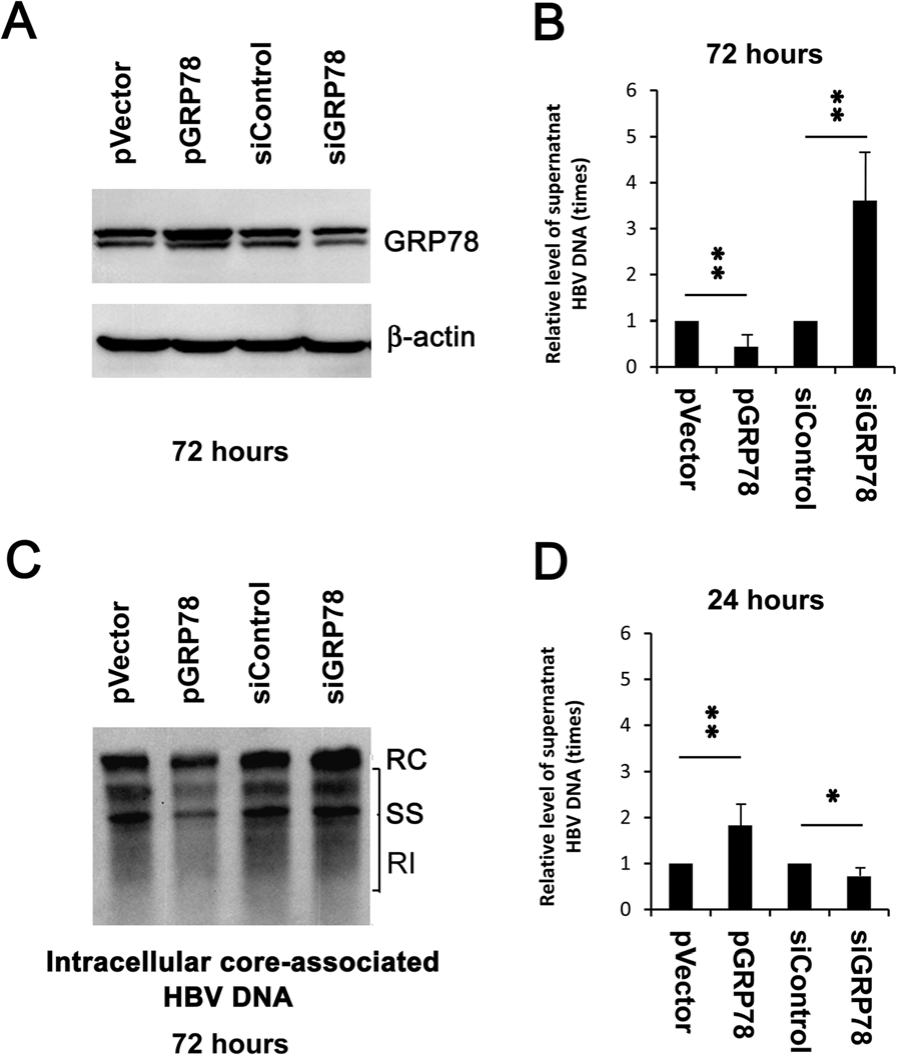
GRP78 mainly inhibited HBV replication and reduced virion production. HepG2.2.15 cells were regularly cultivated and transfected with pGRP78 (GRP78-expressing recombinant plasmid generated from pCMV6-XL5) or siRNA targeted GRP78 (siGRP78) or their controls, pCMV6-XL5 (pVector) and siRNA control (siControl). **a** GRP78, detected as two bands, perhaps due to splice variation, was successfully up-regulated or down-regulated 72 h after transfections. **b** GRP78 overexpression decreased and down-regulation increased the supernatant level of core-associated HBV DNA 72 h after transfections. Results are expressed as n-fold times the supernatant HBV DNA level of test groups over those of controls. **P < 0.01. **c** The similar effect of GRP78 on the intracellular core-associated HBV DNA was observed. Relaxed circular (RC) HBV DNA was the HBV genome, and single stranded (SS) HBV DNA, other bands and the smear were the replication intermediates (RI) of HBV. **d** GRP78 showed an opposite effect on the supernatant level of core-associated HBV DNA 24 h after transfections. Results are expressed as n-fold times the HBV DNA level of test groups over those of controls. *P < 0.05, **P < 0.01

GRP78 has been reported to promote the secretion of HBV particles in HepG2.2.15 cells, but to act as an intracellular anti-HBV factor in HepAD38, a cell line with Tet-off promoter to control its HBV production [22–24]. Though both reports used siRNA to down-regulate GRP78, the decrease and increase in supernatant HBV particles were observed, respectively. This discordance may result from the difference in cell models. Compared with HepAD38 cells, HepG2.2.15 cells produce HBV particles in a continuous manner, which better mimics the persistent infection state of patients. For this reason, HepG2.2.15 cells were used in this study to clarify the overall effect of GRP78 on HBV production. GRP78 was successfully up-regulated and down-regulated by transfections of the recombinant expression plasmid and the siRNA of GRP78, respectively (Fig. 1a). GRP78 overexpression reduced supernatant and intracellular core-associated HBV DNA and GRP78 knockdown showed the opposite effect (Fig. 1b and c). GRP78 was regularly detected as two bands, perhaps due to splice variation. Though the same cell model was used, the effect of GRP78 here was opposite to that reported in the published literature [24], which may result from the uses of different observation time, 72 h in this study and 24 h in literature. To test this possibility, supernatant core-associated HBV DNA was detected 24 h after transfections (Fig. 1d). As result, GRP78 indeed moderately increases the supernatant level of HBV particles. Furthermore, GRP78 was found to inhibit HBV production slightly 48 h and significantly 96 h after transfections. The above results suggest that the HBV replication-inhibiting effect of GRP78 time-dependently overwhelmed its viral secretion-promoting effect and that GRP78 mainly inhibits HBV production in patients with persistent infection.

### GRP78 may not inhibit HBV replication by the IFN-β1-dependent pathway in HepG2.2.15 cells

**Fig. 2:**
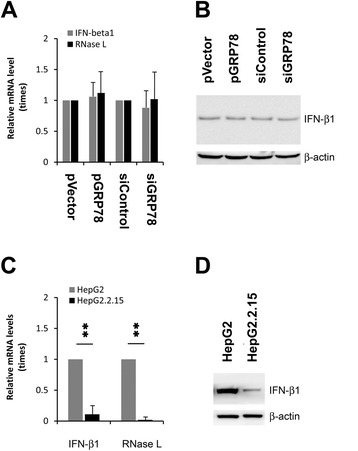
GRP78 did not make use of the IFN-β1-RNase L pathway in HepG2.2.15 cells. HepG2.2.15 cells were regularly cultivated and transfected with pGRP78 (GRP78-expressing recombinant plasmid generated from pCMV6-XL5) or siRNA targeted GRP78 (siGRP78) or their controls, pCMV6-XL5 (pVector) and siRNA control (siControl). **a** GRP78 regulation did not significantly affect the mRNA levels of IFN-β1 and RNase L genes. Results are expressed as n-fold times the IFN-β1 or RNase L level of test groups over those of controls. **b** GRP78 regulation did not significantly affect the protein level of IFN-β1. **c** The mRNA levels of IFN-β1 and RNase L genes in HepG2.2.15 cells were much lower than those in HepG2 cells (parent cells of HepG2.2.15 cells). Results are expressed as n-fold times the IFN-β1 or RNase L level of test groups over those of controls. **P < 0.01. **d** The protein level of IFN-β1 in HepG2.2.15 cells was much lower than that in HepG2 cells

GRP78 reduced HBV production by inhibiting viral DNA replication (Fig. 1). However, the underlying mechanism in HepG2.2.15 cells is unclear yet. Based on the findings in HepG2 cells (parent cells of HepG2.2.15), GRP78 has been reported to inhibit HBV replication through the activation of the IFN-β1-2′,5′-oligoadenylate synthetase-RNase L pathway [22]. Here, we wonder whether GRP78 similarly inhibits HBV replication in HepG2.2.15 cells. Unexpectedly, GRP78 overexpression or knockdown did not significantly affect the mRNA levels of IFN-β1 and RNase L genes (Fig. 2a) or the protein level of IFN-β1 (Fig. 2b) in HepG2.2.15 cells. To clarify the reasons behind the deviation in HepG2.2.15 cells, we further compared the expression levels of those genes of interest between these two cell lines. As results, the mRNA levels of IFN-β1and RNase L genes in HepG2.2.15 cells were only about 1/10 and 1/100 of those in HepG2 cells, respectively (Fig. 2c). Similarly, the protein level of IFN-β1 in HepG2.2.15 cells was much lower than that in HepG2 cells (Fig. 2d). These results ascertain that the IFN-β1-dependent pathway has been blocked and GRP78 does not inhibit HBV replication by this pathway in HepG2.2.15 cells.

### GRP78 decreases HBeAg secretion and increases the retro-transportation of HBeAg precursor from the ER to cytosol

**Fig. 3:**
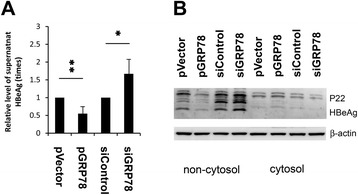
GRP78 promoted the cytosolic retro-transportation of P22 in HepG2.2.15 cells. HepG2.2.15 cells were regularly cultivated and transfected with pGRP78 (GRP78-expressing recombinant plasmid generated from pCMV6-XL5) or siRNA targeted GRP78 (siGRP78) or their controls, pCMV6-XL5 (pVector) and siRNA control (siControl). **a** GRP78 significantly inhibited HBeAg secretion. Results are expressed as n-fold times the supernatant HBeAg level of test groups over those of controls. * *P* < 0.05, **P < 0.01. **b** GRP78 promoted the retro-transportation of HBeAg precursor (P22) from ER to cytosol. Total cytosolic proteins (cytosol) and non-cytosolic proteins (non-cytosol) were separately extracted. The rest bands that varied as P22 or HBeAg are core-related peptides with modification or degradation in different degree

As an ER-resident chaperon protein, GRP78 is reported to specifically co-immunoprecipitate HBeAg precursor and assist its retro-transportation from the ER to the cytosol [12]. In addition, HBeAg precursor in the cytosol is of potential to inhibit HBV replication through formation of encapsidation-incompetent hybrid capsids with core protein [18, 19]. Therefore, GRP78 may inhibit HBV replication by its promoting effect on the cytosolic retro-transportation of HBeAg precursor. In addition, the enhanced retro-transportation of HBeAg precursor may reduces HBeAg synthesis, which is meaningful for HBeAg seroconversion in clinical practice. Indeed, GRP78 overexpression resulted in the increase in cytosolic HBeAg precursor and the decreases in HBeAg secretion (Fig. 3a and b). In contrast, GRP78 knockdown showed the opposite effects. Other bands that varied as P22 or HBeAg are core-related peptides with modification or degradation in different degree [29].

These results further ascertain the anti-HBV effects of GRP78 and suggest that GRP78 inhibits HBV replication by promoting the retro-transportation of HBeAg precursor from the ER to the cytosol in HepG2.2.15 cells.

### GRP78 up-regulation promotes wound healing in HepG2.2.15 cells

**Fig. 4:**
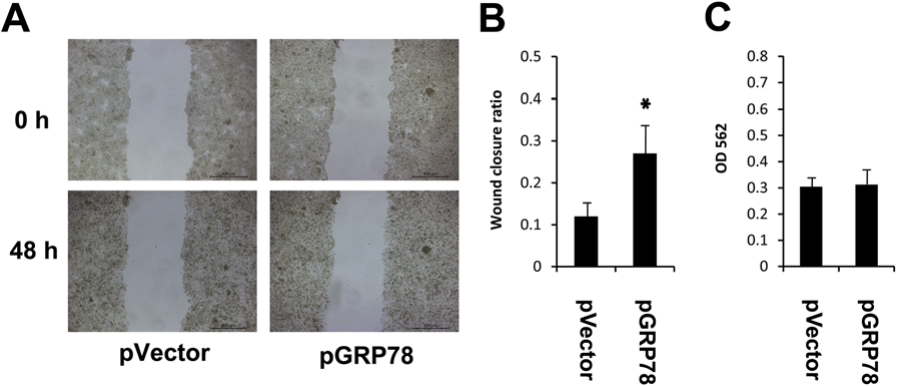
GRP78 overexpression promoted wound healing in HepG2.2.15 cells. HepG2.2.15 cells were regularly cultivated and transfected with pGRP78 (GRP78-expressing recombinant plasmid generated from pCMV6-XL5) or empty vector, pCMV6-XL5 (pVector). **a** After transfection, confluent cells were wounded by sterile pipettes and the wound statuses were photographed at the beginning and 48 h after wounded. **b** Cells with GRP78 overexpression had a significantly higher wound closure ratio. * *P* < 0.05. **c** Cell proliferation was examined using MTT analysis. The proliferation statuses of those cells with and without GRP78 overexpression were identical 48 h after wounded

The above results indicate that GRP78 inhibits both HBV production and HBeAg secretion, correlating with virological and serological responses in clinical practice and suggesting that GRP78 up-regulation as anti-HBV strategy is somewhat better than current viral polymerase-selective inhibitors. However, increasing data imply that GRP78 is involved in the invasion and migration of HCC and other cancers [25, 26]. Since above results show that HepG2.2.15 was unlike other hepatoma cell line in which HBV is inhibited through the IFN-β1-dependent pathway, the cell mobility effects of GRP78 on this cell line was further studied. As results, GRP78 overexpression was found to promote wound healing 48 h after transfection (Fig. 4a and b). This promotion resulted from the enhanced cell mobility rather than the possible effect of GRP78 on cell proliferation since the proliferation statuses of those cells with and without GRP78 overexpression were similar (Fig. 4c).

### GRP78 knockdown enhances HBV replication and HBeAg secretion, but which are abolished by ETV and D6R

**Fig. 5:**
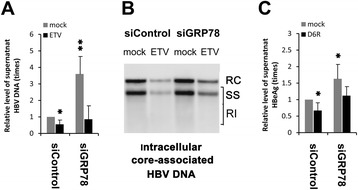
ETV and D6R abolished the enhancing effects of GRP78 down-regulation on HBV replication and HBeAg secretion. HepG2.2.15 cells were regularly cultivated, transfected with siRNA targeted GRP78 (siGRP78) or its control (siControl) and treated with or without ETV (20 nmol/L) or furin inhibitor II (D6R, 100 μmol/L). **a** ETV abolished the enhancing effect of GRP78 down-regulation on supernatant core-associated HBV DNA. Results are expressed as n-fold times the supernatant HBV DNA level of test groups over those of controls. * *P* < 0.05, **P < 0.01. **b**. ETV abolished the enhancing effect of GRP78 down-regulation on intracellular core-associated HBV DNA. Relaxed circular (RC) HBV DNA was the HBV genome, and single stranded (SS) HBV DNA, other bands and the smear were the replication intermediates (RI) of HBV. **c** Furin inhibitor abolished the enhancing effect of GRP78 down-regulation on HBeAg secretion. Results are expressed as n-fold times the supernatant HBeAg level of test groups over those of controls. * *P* < 0.05

HCC is a fatal complication of chronic HBV infection and its prophylaxis of current antiviral options is far from satisfaction [4–8]. Based on its characteristics to inhibit HBV secretion and cancer invasion, GRP78 down-regulation may be a promising supplement for current antiviral options. However, GRP78 down-regulation significantly increased HBV replication and HBeAg secretion (Figs. 1 and 3). Our previous study has shown that ETV, a broadly accepted antiviral drug in clinical practice, combined with D6R, a furin inhibitor that moderately inhibits HBeAg secretion but is unlike the other common furin inhibitor to enhance HBV replication [13], inhibits HBV replication and HBeAg secretion simultaneously [30]. For these reasons, we here examined whether ETV and D6R coped with those unpleasant effects. Fortunately, ETV completely abolished the HBV replication-enhancing effect of GRP78 knockdown though its own effect was moderately reduced (Fig. 5a and b). Similarly, D6R completely abolished the HBeAg secretion-enhancing effect (Fig. 5c).

## Discussion

GRP78 has contradictory effects on HBV life cycle, inhibiting HBV replication and promoting HBV secretion [22–24]. In this study, the overall effect of GRP78 was found to decrease HBV production due to its HBV replication-inhibitory effect time-dependently overwhelming virion secretion-promoting effect in HepG2.2.15 cells, probably due to HBV replication is an upstream event in viral life cycle, implying that GRP78 mainly inhibits HBV replication in patients with persistent infection. Indeed, GRP78 overexpression showed anti-HBV effects, decreases in HBV DNA replication and HBeAg secretion, implying to be favorable for common antiviral goals, undetectable HBV DNA and HBeAg seroconversion in clinical practice. However, GRP78 overexpression increased the mobility of HepG2.2.15 cells based on wound healing tests, which implies the involvement in HCC tumorigenesis and dims the clinical prospect of GRP78 up-regulation. It also suggests that GRP78 knockdown may be favorable for HCC prevention. However, GRP78 knockdown was found to increase HBV replication and HBeAg secretion. Therefore, the above findings imply that GRP78 down-regulation is suitable to serve as a supplementary strategy to improve the HCC prevention efficacy of current antiviral options once the enhancements of HBV replication and HBeAg secretion are effectively blocked.

The mechanism for GRP78 to inhibit HBV replication as well as the secretions of HBsAg and HBeAg is not completely clarified. GRP78 overexpression up-regulates the expressions of IFN-β1, OAS1, OAS2 and RNase L genes and IFN-β1 treatment in return up-regulates the expression of GRP78 in HepG2 cells, implying that the anti-HBV mechanism of GRP78 correlates with the IFNβ1-dependent pathway [22]. However, in this study, we found that HepG2.2.15 cells expressed much less constitutive IFN-β1 and RNase L when compared with HepG2 cells, which is in concordance with the report of other scientists [31, 32]. In addition, GRP78 overexpression or knockdown did not significantly affect the expressions of both mentioned genes. On the other hand, GRP78 was found to promote the retro-transportation of HBeAg precursor from the ER to the cytosol in HepG2.2.15 cell. It is well-documented that cytosolic HBeAg precursor can inhibit HBV replication by formation of HBV encapsidation-incompetent capsids [18, 19]. Therefore, these above results ascertain that the IFN-β1-dependent pathway has been blocked in HepG2.2.15 cell and GRP78 inhibits HBV replication by promoting retro-transportation of HBeAg precursor from the ER to the cytosol. To restore the vigor of the IFN-β1-dependent pathway may be a new strategy to treat CHB in the future.

Immune tolerance is a major factor underlying the maintenance of chronic HBV infection. HBeAg as an immunotolerance inducer plays key roles in the maintenance of immune tolerance as shown by the facts that HBeAg-defective variants rarely cause a de novo chronic infection and that HBeAg modulates host immune responses to HBcAg [14–16]. In this study, GRP78 was found to inhibit HBeAg secretion via enhancement of the retro-transportation of HBeAg precursor, which is meaningful for HBeAg seroconversion, an important treatment endpoint, in clinical practice.

It is well documented that GRP78 facilitates invasions of HCC and other cancers [25–27]. Perhaps due to the persistent existence of HBV, HepG2.2.15 cells was unlike other hepatoma cell line in which HBV is inhibited through the IFN-β1-dependent pathway. However, based on wound healing tests, the effect of GRP78 on the mobility of HepG2.2.15 cells was similar to that of HBV-free cell lines. In addition, GRP78 usually highly expresses in HCC tissues, and the expression level is positively correlates with portal and intra-hepatic invasions [33, 34]. The HCC-contributing mechanisms of GRP78 include alleviating the ER stress, mediating by loss of the tumor suppressor PTEN and enhancing the activation and activity of FAK [35–37]. Thus, it is ascertained that GRP78 is substantially involved in HCC tumorigenesis. Because HCC is a fatal complication of chronic HBV infection, the possibility of GRP78 up-regulation as an anti-HBV strategy seems to be dismissed unless GRP78 up-regulation can lead to the rapid elimination of chronic HBV infection in early stage. Unfortunately, it is well known that the elimination of chronic HBV infection is an ambitious expectation currently.

The HCC prevention efficacy of current antiviral options is urged to improve, especially for patients with liver cirrhosis [4–8]. The on-treatment cumulative HCC rate at 5 years is about 3% in patients with CHB and up to 30% in those with cirrhosis. The on-treatment cumulative HCC rate at 10 years is about 30% in patients with CHB and up to 50% in those with cirrhosis. In view of the effects of GRP78 on HCC, GRP78 down-regulation may be a potentially supplementary measure to improve the HCC prevention efficacy of current antiviral therapy. Indeed, GRP78 knockdown may decrease the invasion capability, reduce the risk of resistance to sorafenib and enhance the adenosine-induced apoptosis [27, 38, 39]. However, some articles report the opposite effect. GRP78 down-regulation enhances migration ability in some cell lines of HCC, perhaps due to their unique pattern of GRP78 expression [40, 41]. In addition, GRP78 down-regulation enhanced HBV replication and HBeAg secretion though these unpleased effects were abrogated by ETV and furin inhibitor, respectively. Therefore, GRP78 as a therapeutic target is of potential, but has substantial limitations, which urges for more intensive studies in the future.

## Conclusions

Although it mainly demonstrates anti-HBV effects, GRP78 is not a suitable anti-HBV target due to its potential risk of HCC invasion. Fortunately, GRP78 down-regulation is of potential to be an additive strategy to cover the HCC prevention shortage of current antiviral therapy.

## Methods

### Plasmid constructs and siRNA synthesis

GRP78-expressing recombinant plasmid, pGRP78, and its original plasmid pCMV6-XL5 (named as pVector in this study) were purchased from OriGene Technologies, Rockville, MD, USA. The small interfering RNA (siRNA) against GRP78, siGRP78, (5′-CCAAG AUGCU GACAU UGAA dTdT-3′) and the negative control siRNA against the severe acute respiratory syndrome coronavirus, siControl, (5′-GCACU UGUCU ACCUU GAUG dTdT-3′) were synthesized by RiboBio Corporation, Guangzhou, China.

### Cell culture and transfections

HepG2 and HepG2.2.15 cells were grown in Dulbecco’s modified Eagle’s medium with 10% fetal bovine serum. For HepG2.2.15 cells, the medium was supplemented with 380 μg/mL geneticin. HepG2.2.15 cells were transfected with 1.6 μg/well pGRP78 or pVector and 200 nmol/L siGRP78 or siControl, respectively. All transfections were performed in 12-well plates suing Lipofectamine 2000 (Invitrogen Corporation, Shanghai, China) according to the manufacturer’s instructions. To abolish the effects of GRP78 down-regulation on HBV replication or HBeAg secretion, cells were treated with 20 nmol/L ETV (Sigma-Aldrich Corporation, St. Louis, MO, USA) or 100 μmol/L D6R (EMD Biosciences, La Jolla, CA, USA), respectively. To evaluate HBV replication and GRP78 expression, cells were harvested 72 h after transfection unless otherwise indicated.

### Detections of GRP78, IFN-β1 and intracellular HBV antigens

For the detection of GRP78 and IFN-β1, total cellular proteins were prepared by lysing the cells on ice for 30 min in lysis buffer (50 mmol/L Tris, pH 7.4, 150 mmol/L NaCl, 1% NP-40, 1% SDS, 1 mmol/L phenylmethylsulfonyl fluoride, 10 mg/L aprotinin, 10 mg/L leupeptin). For the detections of intercellular HBcAg, HBeAg and HBeAg precursor the cytosolic and non-cytosolic cellular proteins were separately extracted as reported [12]. Briefly, after cell membrane was cautiously broken on ice by a digitonin buffer (50 mmol/L Tris, pH 8, 150 mmol/L NaCl, 22.5 mg/L digitonin, 0.5 mmol/L phenylmethylsulfonyl fluoride, 1 mg/L aprotinin, 1 mg/L leupeptin), the cytosolic fraction of cell lysates including soluble cellular proteins and the non-cytosolic fraction including the cellular proteins of remaining cell debris (nucleus, endoplasmic reticulum and Golgi complex) were extracted independently. Sampling was normalized to total cellular, cytosolic or non-cytosolic proteins. The total and sorted cellular proteins were separated and transferred onto polyvinylidene fluoride membranes (Millipore Corporation, Billerica, MA, USA) using standard techniques. Immunoblot analysis was performed using polyclonal antibodies to human GRP78 (Novus Biologicals, Littleton, CO, USA), IFN-β1(Novus Biologicals,) or HBcAg (DAKO, Carpinteria, CA, USA) and enhanced chemiluminescence reagent (Invitrogen Corporation, Shanghai, China).

### Detections of supernatant and intracellular core-associated HBV DNA

The isolations of supernatant and intracellular core particles were performed as reported [42]. The core-associated HBV DNA was extracted using phenol-chloroform after digested with 60 mg/L of proteinase K at 55 °C for 1 h. The supernatant core-associated HBV DNA was quantitatively examined using real-time fluorescent polymerase chain reaction (PCR) (Taan Gene Company, Guangzhou, china). The intracellular core-associated HBV DNA was detected using Southern blot analysis. Sampling was balanced based on the protein level in cell lysate. The isolated DNA was separated and transferred onto nylon membranes (Roche Applied Science, Indianapolis, IN, USA). After hybridized with digoxigenin-labeled DNA probes, all membranes were incubated with horseradish peroxidase-labeled anti-digoxigenin antibody (Roche Applied Science), and developed with an enhanced chemiluminescence reagent (Invitrogen Corporation).

### Quantitative detections of the mRNA levels of IFN-β1 and RNase L genes

The cellular total RNA was extracted using TRIzol (Invitrogen Corporation) according to the manufacturer’s instructions. After DNase I (Ambion, Austin, TX, USA) treatment and quantification by photospectrometry, RNA purity was assessed by electrophoresis on 1% agarose gels, and then 1 ng of total RNA was reverse transcribed in a 20 μl reaction (Promega, Madison, WI, USA) according to the kit manufacturer’s protocol. The relative mRNA levels of IFN-β1, RNase L and glyceraldehyde-3-phosphate dehydrogenase (GAPDH, housekeeping control) were quantified using SYBR Green Real-Time PCR Kits (Takana Bio, Otsu, Japan). The primers were as follows: IFN-β1 (211 bp), forward 5′- GACCA ACAAG TGTCT CCTCC AAA -3′, reverse 5′- GAACT GCTGC AGCTG CTTAA TC -3′; RNase L (141 bp), forward 5′- TTGAG GCGAA AGACA AAGGA G -3′, reverse 5′- GTCAC AGGCG TTTAC ATCTG C -3′; and GAPDH (138 bp), forward 5′-GCACC GTGAA GGCTG AGAAC-3′, reverse 5′-TGGTG AAGAC GCCAG TGGA-3′. The reaction was performed using 2 μl of reverse transcription product for each sample, and three runs of real-time PCR were performed for every sample. Amplification was conducted at 95 °C for 5 s, and an annealing temperature of 60 °C for 31 s over 40 cycles. At the end of each program, melting curve analysis was carried out. Data for IFN-β1and RNase L were normalized using expression of the housekeeping gene GAPDH in order to ensure comparability.

### Detection of supernatant HBeAg

HBeAg in media was quantified using commercial kits of chemiluminescence immunoassay (USCNK Life Science Incorporation, Wuhan, China).

### Wound healing and cell proliferation analyses

Cell monolayer with or without GRP78 overexpression was carefully wounded by sterile pipette. After removing the debris by 3 times of washes with PBS, the monolayer was cultured in Dulbecco’s modified Eagle’s medium containing 0.5% fetal bovine serum for 48 h. Photographs (by microscope, × 100) were taken immediately after wound incision and at the time point of 48 h. Would closure ratio = (initial width - later width)/initial width. Ten would closure ratios at different locations were calculated for each monolayer. For cell proliferation assay, 500 μl sterilized 3-(4,5-dimethyl-2-thiazolyl)-2,5-diphenyl-2-H-tetrazolium bromide (MTT) solution (0.5 mg/ml) were added into each well and the cells were incubated for 4 h in normal culture condition. The absorbance at 562 nm was measured using microplate reader after the crystallization was dissolved by 500 μl dimethylsulfoxide.

### Statistical analysis

The differences in supernatant HBV DNA and HBeAg, the mRNA levels of IFN-β1 and RNase L, wound closure ratios and cell proliferations were analyzed using the Student’s *t*-test based on the data from three independent experiments that were normally consisted of three independent tests each. A *P* < 0.05 was considered statistically significant. All statistical analyses were conducted using SPSS software (version 11; SPSS Incorporation, Chicago, IL, USA).

## Abbreviations

CHB: Chronic hepatitis B
D6R: Hex-D-arginine
ER: Endoplasmic reticulum
ETV: Entecavir
GAPDH: Glyceraldehyde-3-phosphate dehydrogenase
GRP78: Glucose-regulated protein
HBcAg: Hepatitis B core antigen
HBeAg: Hepatitis B e antigen
HBsAg: Hepatitis B surface antigen
HBV: Hepatitis B virus
HCC: Hepatocellular carcinoma
IFN: Interferon
MTT: 3-(4,5-dimethyl-2-thiazolyl)-2,5-diphenyl-2-H-tetrazolium bromide
PCR: Polymerase chain reaction
siRNA: Small interfering RNA
